# Dopaminergic Meso-Cortical Projections to M1: Role in Motor Learning and Motor Cortex Plasticity

**DOI:** 10.3389/fneur.2013.00145

**Published:** 2013-10-07

**Authors:** Jonas A. Hosp, Andreas R. Luft

**Affiliations:** ^1^Clinical Neurorehabilitation, Department of Neurology, University of Zurich, Zurich, Switzerland; ^2^Rehabilitation Initiative and Technology Center Zurich, RITZ, Zurich, Switzerland; ^3^Department of Neurology, Johns Hopkins University, Baltimore, MD, USA

**Keywords:** dopamine, motor cortex, motor learning, cortical plasticity, memory

## Abstract

Although the architecture of a dopaminergic (DA) system within the primary motor cortex (M1) was well characterized anatomically, its functional significance remained obscure for a long time. Recent studies in rats revealed that the integrity of DA fibers in M1 is a prerequisite for successful acquisition of motor skills. This essential contribution of DA for motor learning is plausible as it modulates M1 circuitry at multiple levels thereby promoting plastic changes that are required for information storage: at the network level, DA increases cortical excitability and enhances the stability of motor maps. At the cellular level, DA induces the expression of learning-related genes via the transcription factor c-Fos. At the level of synapses, DA is required for the formation of long-term potentiation, a mechanism that likely is a fingerprint of a motor memory trace within M1. DA fibers innervating M1 originate within the midbrain, precisely the ventral tegmental area (VTA) and the medial portion of substantia nigra (SN). Thus, they could be part of the meso-cortico-limbic pathway – a network that provides information about saliency and motivational value of an external stimulus and is commonly referred as “reward system.” However, the behavioral triggers of the release of dopamine in M1 are not yet identified. As alterations in DA transmission within M1 occur under various pathological conditions such as Parkinson disease or ischemic and traumatic brain injury, a deeper understanding of the interaction of VTA/SN and M1 may reveal a deeper insight into a large spectrum of neurological disorders.

## Introduction

The primary motor cortex (M1) receives dopaminergic (DA) projections from mesencephalic brainstem nuclei. The integrity of this DA meso-cortical pathway has been recently shown to be a prerequisite of successful motor learning in rats. Apart from providing details on morphology and behavioral experiments, we describe how DA signaling facilitates plastic changes within M1 circuitry at various levels thereby promoting storage of newly acquired movement sequences. In addition to putative behavioral triggers and dynamics of DA release in M1, the assignability of knowledge gained in rodent models to human subjects becomes discussed. Finally, we provide an overview of M1 plasticity in neurological diseases characterized by a DA deficit.

## DA in M1 is Necessary for Motor Learning in Rats

Apart from prefrontal region (PFC), sensorimotor areas receive the largest amount of DA innervation within the rodent neocortex (1). In M1, DA terminals are distributed inhomogeneously with a preference for deep cortical layers [layer V/VI (2)]. Regarding postsynaptic elements, D1 receptors are expressed in both, superficial (layers I, II, IIIa) and deep (V and VI) layers (3), whereas D2-receptors are expressed primarily in layer V but 10-fold less than D1 (4). Additionally, co-localization of both receptor subtypes were observed in layer V/VI motor neurons (5). Retrograde tracing identified the ipsilateral DA midbrain nuclei to be the origin of motor-cortical DA innervation: about 73% of DA midbrain neurons projecting to M1 are located in the rostro-lateral ventral tegmental area (VTA, also referred to as region A10) whereas a smaller amount of neurons can be found in the rostro-medial substantia nigra [SN; also referred to as region A9; 12%; (6)].

To define the functional role of this DA system, we assessed the effect of removing DA terminals within M1 on acquisition of a motor skill-learning paradigm (6, 7). Rats were trained in a single-pellet reaching task (8), while selective destruction of DA terminals was performed by injecting 6-hydroxi-dopamine (6-OHDA) either directly in M1 or within the rostral VTA/SN, both contralateral to the preferred paw. DA depletion of M1 resulted in an impaired gain in motor performance between subsequent training sessions (inter-session learning) when compared to controls. This impairment could be resolved by substituting levodopa directly within M1 using osmotic mini-pumps. As the short-term improvement of performance within one session (intra-session learning) was not affected by lesions, DA seems to be required for longer-lasting storage mechanisms underlying motor memory consolidation (9). Inducing 6-OHDA lesions in rats that already learned the task did not influence reaching performance, indicating a role of DA for movement acquisition but not for movement execution. As the effect of 6-OHDA lesions could be mimicked by injection of the D1-receptor antagonist SCH-29930 or the D2-receptor antagonists raclopride and sulpiride, motor learning seems to depend on both receptor subtypes (7). Whereas direct 6-OHDA lesions in M1 resulted only in a gradual but significant impairment of motor skill acquisition, destroying the origin of the motor-cortical DA innervation in VTA/SN completely abolished motor learning. This can be explained by a higher effectiveness of DA depletion caused by brainstem lesions when compared to direct injection of 6-OHDA into M1. Remarkably, 6-OHDA lesions within the midbrain could be applied with sufficient precision to avoid any side effects like motivational deficits or extrapyramidal symptoms that would be expected in case of damaging projections to the PFC or to striatum. In summary, the integrity of DA projection from midbrain to M1 is a prerequisite of successful movement acquisition based on D1- and D2-receptor mediated mechanisms.

## How DA May Promote Plasticity in M1

Although extra-cortical brain regions as cerebellum and basal ganglia contribute to motor learning (10), M1 is thought to be the place where motor memory becomes stored (11). This motor memory storage depends on M1 ability to undergo experience-dependent changes, a phenomenon commonly referred to as motor plasticity (12). DA has been shown to modulate M1 circuitry at several levels thereby affecting various processes of motor-learning dependent plasticity.

### Level of M1 network-physiology

Motor learning induces an enlargement of the motor-cortical representation (motor map) of particular body-parts that became trained, a phenomenon observed in rodents, primates, and humans (13–15). This enlargement is learning specific as it does not occur in response to mere motor activation and its magnitude is proportional to learning success (16, 17). The enlargement of motor representations furthermore depends on intact signaling of modulatory neurotransmitters: preventing cholinergic transmission by destroying basal forebrain structures in rats abolished both, expansion of motor maps and skill acquisition (18). Comparably, intra-motor-cortical injection of the D2-receptor antagonist raclopride induced a collapse of motor representations evoked by epidural electrical microstimulation in rats, whereas blocking D1 receptors with SCH 23390 had no effects (19).

Besides the spatial expansion of motor maps, transcranial magnetic stimulation (TMS) in humans revealed a training-related increment in M1 excitability in response to learning a piano sequence (15). In rats, blocking D2-receptors by intra-cortical injection of raclopride significantly increased stimulation thresholds necessary to evoke motor responses indicating a decreased level of motor-cortical excitability (19). Thus, by stabilizing motor representations and by increasing cortical excitability via D2-receptor dependent processes, DA signaling in M1 seems to be ideally suited to support learning-related changes on M1 network level.

### Level of gene expression

Motor learning requires protein synthesis within M1 neurons (20) as a prerequisite of subsequent learning-dependent changes including increased spine-turnover and dendritic growth (11, 12). In M1 of rats, expression of the transcription factor c-Fos becomes induced in response to learning an acrobatic skill (21). Because c-Fos expression was highest during skill acquisition and subsequently decreased in the maintenance phase, the learning-specificity of this phenomenon is highly plausible. c-Fos is considered to be not only a marker of recent neuronal activation but also for experience-dependent changes (22) and is known to become induced by DA signaling (23). Electrical stimulation of neurons within the rostro-lateral VTA induces an increased expression of c-Fos within the ipsilateral M1 that can be blocked by intra-cortical injection of the D1- and D2-receptor antagonists SCH 23990 and raclopride (6). Thus, DA efferents from the midbrain are capable to support the expression of learning-relevant proteins in M1.

### Level of synaptic transmission

In rats, motor skill learning induces a long-lasting increase of synaptic strength in M1 horizontal connections of layer II/III suggesting an association with long-term potentiation (LTP)-like plasticity (24). In line with this assumptions, capacity to induce LTP within these connections was reduced whereas long-term depression (LTD) was increased, suggesting that the learning-induced gain in synaptic strength expended the capacity of LTP-formation (25). Several weeks after skill acquisition, the ability to form LTP was restored while the horizontal connections of layer II/III remained strengthened (26). Bath application of both, the D1-receptor antagonist SCH 29339 and the D2-receptor antagonist raclopride markedly reduced the ability of M1 horizontal connections to form LTP (7), suggesting the necessity of intact DA signaling for long-lasting synaptic plasticity in M1.

### DA outside of M1

As tetanic stimulation of somatosensory cortex (S1) mediates LTP induction in M1 (27), it is assumed that motor-learning dependent synaptic plasticity is mediated by somatosensory input (28, 29). Thus, a close and precise interaction between M1 and S1 is a prerequisite for successful motor learning (28). In S1, intra-cortical injection of both, D1- and D2-receptor antagonists induced an enlargement of somatosensory evoked potential (SEP)-amplitude consistent with an increased cortical excitability (30). Thus, by reducing S1 excitability, DA may serve focusing on relevant (= strong) somatosensory input thereby improving signal-to-noise ratio (31) and improving sensory discrimination (32). However, if improved somatosensory information processing in sensorimotor cortex could also be one mechanism by which DA facilitates motor learning remains to be tested.

## What Do Dopaminergic Signals in M1 Encode?

Dopaminergic neurons projecting to M1 are located in the rostro-lateral VTA and, to a lesser extent, in the rostro-medial SN (6). Thus, this projection is part of the meso-cortico-limbic system that provides DA input to cortical (mainly prefrontal cortex) and limbic (e.g., amygdala and hippocampus) structures (33, 34). In general, this system is thought to evaluate environmental stimuli with respect to their value and behavioral significance. Whereas the activity of specific neuronal subgroups is coupled to a particular content like motivational value or saliency (35), the time course of dopamine release may encode if a stimulus is pleasant (“rewarding”) or aversive [“punishing”; (36, 37)]. In rodents, DA projections to PFC critically modulate spatial working memory (38, 39) and attention selection (40). In a computational model that simulates the effect of DA on PFC network (39), low concentrations of DA constitute a “gate closed” state characterized by a boosted inhibitory drive and stereotypical behavior. At higher concentrations, DA induces a “gate open” state characterized by an increased excitability of the network and consecutive explorative behavior. Based on these considerations, one may hypothesize that the DA midbrain-to-M1 projection facilitates the occurrence of plastic changes within the motor-cortical network in response to salient, novel, or appetitive stimuli. However, it is illusive which environmental cues during motor learning are capable of activating midbrain neurons that trigger the release of DA in M1.

In contrast to DA neurons forming the nigro-striatal pathway, meso-cortically projecting neurons are characterized by fast firing (30 Hz), slow dopamine reuptake and reduced if not lacking D2-receptor dependent auto-inhibition (41). This class of DA cells therefore seems to be well suited to provide a sustained release of DA. In addition, voltammetric studies revealed that the clearance of extracellular DA within the amygdala or the PFC is considerably slower when compared to striatum (42, 43). Thus, DA projections to cortical and limbic structures rather seem to influence DA concentrations on a larger time-scale. This tonic form of modulatory neurotransmission would be well suited to support longer-lasting changes required for motor memory consolidation such as LTP-like plasticity. However, the kinetics of DA release and clearance within M1 still remain to be experimentally established.

## Can These Findings be Assigned to Humans?

Notable differences in magnitude and organization have to be taken into account between species regarding DA innervation. At first, the midbrain of primates harbors three to seven times more DA neurons when compared to rodents (44). Thus, cortical – and especially motor-cortical – DA innervation is accordingly much more pronounced in primates (45, 46). Furthermore, cortically projecting DA neurons in primates can be found beyond VTA and SN: in owl and rhesus monkeys, 15% of DA efferents toward M1 are located within the retrorubral field [RRF, also referred to as region A8; (2, 47)], a region that completely lacks meso-cortically projecting neurons in rodents (48, 49). Finally, whereas DA terminals are homogenously distributed among cortical layers in primates, deeper layers receive stronger DA innervation in rodents than superficial ones (2, 50). Taken together, presumably as a consequence of neocortical development, DA innervation of cortex underwent a gain in complexity during phylogeny. However, no significant differences in quantity and distribution of DA terminals in M1 could be detected for non-human primates and humans (51).

Despite these pronounced differences between species, DA signaling in humans seems to have similar function for motor learning when compared to the rodent model: in humans, administration of a single dose of levodopa or the D2-receptor agonist cabergoline facilitated the encoding of an elementary motor memory in M1 [influencing the direction of TMS – evoked thumb movement by prior training; (50, 52)]. On the other hand, blocking DA-receptors with haloperidol interfered with motor learning (52). The magnitude of this levodopa-induced improvement in movement acquisition depends on multiple factors like age (50, 53) and genetic variations of DA metabolizing enzymes and DA receptor isoforms (54). Thus, a beneficial effect of external DA stimulation is mainly expectable when the DA system is challenged due to degenerative processes associated with (healthy) aging or genetic polymorphisms related to a low effectiveness of DA transmission.

Although the cited studies in humans are in a good agreement with findings derived from animal research, transfer of knowledge across species is limited due to differences in experimental methodology and the increased complexity of the DA system in higher-order species. For example, intra-cortical injection of the D2-receptor antagonist raclopride reduces M1 excitability in rats (19) whereas administration of DA antagonists in humans has the opposite effect (52, 55, 56). However, as intra-cortical injection in the animal model is expected to induce only local changes, systemic application of drugs will influence several systems thereby causing a different net effect. In this example, blocking striatal DA-receptors may disinhibit excitatory thalamo-cortical projections thereby increasing M1 excitability (57, 58). Furthermore, the effect of exogenous DA stimulation on motor learning in humans depends on various genetic polymorphisms within cleaving enzymes (e.g., catechol-*O*-methyltransferase), receptor isoforms and reuptake transporters (54). Thus, administration of levodopa may range from beneficial to disadvantageous effects depending on the individual genetic profile. In contrast to humans, genetic profiles in rodent inbred stems are expected to be quite homogenous. Thus, it is not surprising that results in human studies spread around a larger range when compared to animal experiments.

## The Role of DA for M1 Plasticity in Neurological Diseases

In Parkinson disease (PD), degeneration of DA neurons projecting to the neocortex occurs early in the course of disease (59, 60) and leads to a 70% reduction of DA fibers within M1 and other frontal cortical areas (61). Apart from the loss of nigro-striatal DA projections causing the classical extrapyramidal symptoms of PD like stiffness, tremor, and bradykinesia (62), PD patients also suffer from motor-learning deficits (63, 64). In line with these observations, capability of M1 to undergo plastic changes in response to TMS paired associative stimulation or theta-burst stimulation protocols is abolished in PD patients off DA medication (65–69). Interestingly, substitution of a single dose of l-DOPA rescued LTP- (and LTD)-like plasticity only in patients on chronic DA medication (65, 67, 68) but not in newly diagnosed PD patients naïve to l-DOPA (66). Thus, restoration of M1 plasticity likely reflects a long-duration effect of l-DOPA treatment, whereas reduction of extrapyramidal symptoms that occurs immediately after l-DOPA substitution depends on a short-duration response (65). In PD patients with a long-lasting course of disease suffering from fluctuations (e.g., wearing off phenomenon) or dyskinesia (e.g., peak of dose dyskinesia) l-DOPA dependent restoration of M1 plasticity is deficient (67) and even dysfunctional effects on plasticity-formation occur in response to l-DOPA administration (65). Taken together, M1 capability for plasticity-formation in PD patients depends on duration of disease (= degree of denervation) and persistent effects of DA treatment. If these phenomena can be explained by the degeneration of DA fibers directly within M1, by indirect effects on M1 circuitry (e.g., changes in striato-thalamo-cortical signaling) or by a sum effect of both is still an open question.

Apart from PD where DA neurons irreversibly degenerate, a state of a functional “DA deficit” may also emerge in case of brain injury (53, 54). After experimental application of traumatic brain lesions in rats, a sustained down-regulation of DA reuptake transporters (DAT) and increased concentrations of the DA synthesizing enzyme tyrosine hydroxylase (TH) within the frontal cortex indicate profound alterations of DA transmission in response to mechanical damage (70, 71). If ischemic brain injury leads to similar widespread changes in DA signaling has not been investigated, yet. However, chronic stroke survivors also profited from l-DOPA administration in acquiring an elementary motor-learning paradigm (72) and levodopa treatment in combination with physiotherapy was superior to physiotherapy alone in hemiparetic stroke patients (73, 74).

## Conclusion and Future Research

In rats, meso-cortical DA projections are required for successful motor learning (Table 1: 1–3). By promoting plasticity at the level of learning-related gene expression, synaptic transmission, and network-physiology, DA signaling is well suited to facilitate the storage of novel movement sequences within M1 circuitry. Based on present literature, we propose that mesencephalic DA neurons innervating M1 are activated by novel and salient external stimuli, exerting a tonic and longer-lasting shift in cortical DA concentrations. Thus, the DA meso-cortical pathway is thought to support M1 circuitry to adapt to changes in environmental requirements. However, this hypothesis needs further experimental confirmation: combining telemetric recording of VTA activity and M1 field potentials during a skilled reaching task could reveal the behavioral cue preceding the activation of M1-projecting mesencephalic neurons. Furthermore dynamics of DA signaling within M1 could be assessed using voltammetric measurements.

**Table 1:** **Collection of key papers studying the effect of dopaminergic signaling on plasticity in M1 and/or motor learning**.

Reference	Species	Effect of DA regarding M1 plasticity/motor learning
1. Molina-Luna et al. (7)	Rat	DA signaling in M1 is required for successful motor learning
		Blocking D1- and D2-receptors in M1 interferes with motor learning
		Blocking D1- and D2-receptors interferes with LTP-formation in M1
2. Hosp et al. (19)	Rat	Blocking D2-receptors in M1 induces a breakdown of motor representations
		Blocking D2-receptors reduces excitability of M1 network
3. Hosp et al. (6)	Rat	Dopaminergic projections to M1 originate in the ipsilateral VTA (and SN)
		The integrity of this meso-cortical pathway is essential for motor learning
		VTA-stimulation induces expression of learning-relevant genes in M1
4. Floel et al. (50)	Humans (healthy)	l-DOPA facilitates encoding of motor memory in M1 (TMS-evoked thumb movements) in elderly but not in young subjects
5. Meintzschel and Ziemann (52)	Humans (healthy)	DA agonist cabergoline facilitates encoding of motor memory (TMS-evoked thumb movements) in M1
		DA antagonist haloperidol enhances excitability of M1
6. Floel et al. (53)	Humans (healthy)	l-DOPA facilitates skilled motor learning in elderly but not in young subjects
7. Pearson-Furhop et al. (54)	Humans (healthy)	Effect of external dopaminergic stimulation depends on the individual profile of polymorphisms in DA-related genes
8. Morgante et al. (67)	Humans (PD)	Plasticity-inducing TMS protocols have no effect in M1 of PD patients
		l-DOPA restores M1 capability to undergo plastic changes except in patients suffering from dyskinesia
9. Ueki et al. (68)	Humans (PD)	Plasticity-inducing TMS protocols have no effect in M1 of PD patients
		l-DOPA restores M1 capability to undergo plastic changes
10. Kishore et al. (65, 66)	Humans (PD)	Plasticity-inducing TMS protocols have no effect in M1 of PD patients
		l-DOPA-dependent restoration of plasticity in M1 capability is a long-term effect
		l-DOPA has no or even disadvantageous effects in patients suffering from fluctuations/dyskinesia
11. Scheidtmann et al. (73)	Humans (stroke)	l-DOPA in combination with physiotherapy facilitates motor recovery in stroke patients
12. Floel et al. (72)	Humans (stroke)	l-DOPA facilitates encoding of motor memory in M1 (TMS-evoked thumb movements) in stroke patients

PD, Parkinsons disease; SN, substantia nigra; TMS, transcranial magnetic stimulation; VTA, ventral tegmental area.

In human subjects, the specific role of the meso-cortical projections toward M1 can be hardly defined, as alterations (systemic administration of drugs or degeneration of DA neurons) usually affect the entire DA system. However, DA signaling seems to influence M1 plasticity and motor learning in a quite similar way when compared to the rodent model: administration of l-DOPA improves the encoding of elementary motor memory in healthy subjects characterized by a low effectiveness of DA signaling (e.g. due to aging process; Table 1: 4–6). In patients suffering from PD, induction of plastic changes using TMS is severely impaired, a phenomenon that can be reversed by administration of l-DOPA at least in the early course of disease (Table 1: 8–10). In future research on humans, special attention should be paid to genetic variability with respect of DA receptor isoforms and DA cleaving or metabolizing enzymes as this molecular profile substantially shapes the effect of exogenous DA stimulation (Table 1: 7).

Apart from PD where l-DOPA substitution is the medication of choice, a DA deficiency requiring intervention may also occur in case of ischemic (or traumatic) brain injury (Table 1: 11–12). To test this hypothesis, changes on key determinants of DA signaling could be assessed in response to experimentally applied ischemic lesions (M1) in rats with respect to peri-infarct cortex and target regions of the DA system remote to the lesion side. Besides the valuable insight into long-term consequences of ischemic stroke on modulatory transmitter systems, these experiments could provide strong support for the application of l-DOPA in stroke survivors highlighting the requirement for further clinical trials.

## Conflict of Interest Statement

The authors declare that the research was conducted in the absence of any commercial or financial relationships that could be construed as a potential conflict of interest.
